# Virotherapy as Potential Adjunct Therapy for Graft-Vs-Host Disease

**DOI:** 10.1007/s40139-018-0186-6

**Published:** 2018-11-19

**Authors:** Nancy Y. Villa, Grant McFadden

**Affiliations:** Biodesign Center for Immunotherapy, Vaccines and Virotherapy, Arizona State University, Tempe, AZ 85287 USA

**Keywords:** Virotherapy, Myxoma virus, Allogeneic transplantation, Hematologic malignancies, GvHD, GvT

## Abstract

**Purpose of Review:**

This review discusses the pathophysiology, risk factors, and the advances in the prevention or treatment of graft-vs-host disease (GvHD) by exploiting adjunct virotherapy. In addition, nonviral adjunct therapeutic options for the prevention of GvHD in the context of allogeneic hematopoietic stem cell transplantation (allo-HSCT) are discussed. The role of oncolytic viruses to treat different HSCT-eligible hematological cancers is also considered and correlated with the issue of GvHD in the context of allo-HSCT.

**Recent Findings:**

Emerging therapies focused on the prevention or treatment of GvHD include the use of regulatory T cells (Tregs), mesenchymal stem cells (MSCs), microbiome manipulation, B cell inhibitors, among others. Our lab and others have reported that an oncolytic DNA virus from the *Poxviridae* family, called myxoma virus (MYXV), not only exhibits oncolytic activity against various hematologic malignancies like multiple myeloma (MM) or acute myeloid leukemia (AML) but also, in addition, ex vivo MYXV treatment of human allogeneic-bone marrow transplants (allo-BMT), or allo-peripheral blood mononuclear cell (allo-PBMC) transplants can abrogate GvHD in xenografted mice without impairing graft-vs-tumor (GvT) effects against residual cancer. To date, this is the first and the only oncolytic virus with a dual potential of mediating oncolysis against a residual cancer target and also inhibiting or preventing GvHD following allo-HSCT.

**Summary:**

This review discusses how oncolytic virotherapy can be applied as a potential adjunct therapy for the potential treatment of GvHD. In addition, we highlight major emerging nonviral therapies currently studied for the treatment or prevention of GvHD. We also review the emerging oncolytic virotherapies against different hematological cancers currently eligible for allo-HSCT and highlight the potential role of the oncolytic virus MYXV to decrease GvHD while maintaining or enhancing the positive benefits of GvT.

## Introduction

**Fig. 1:**
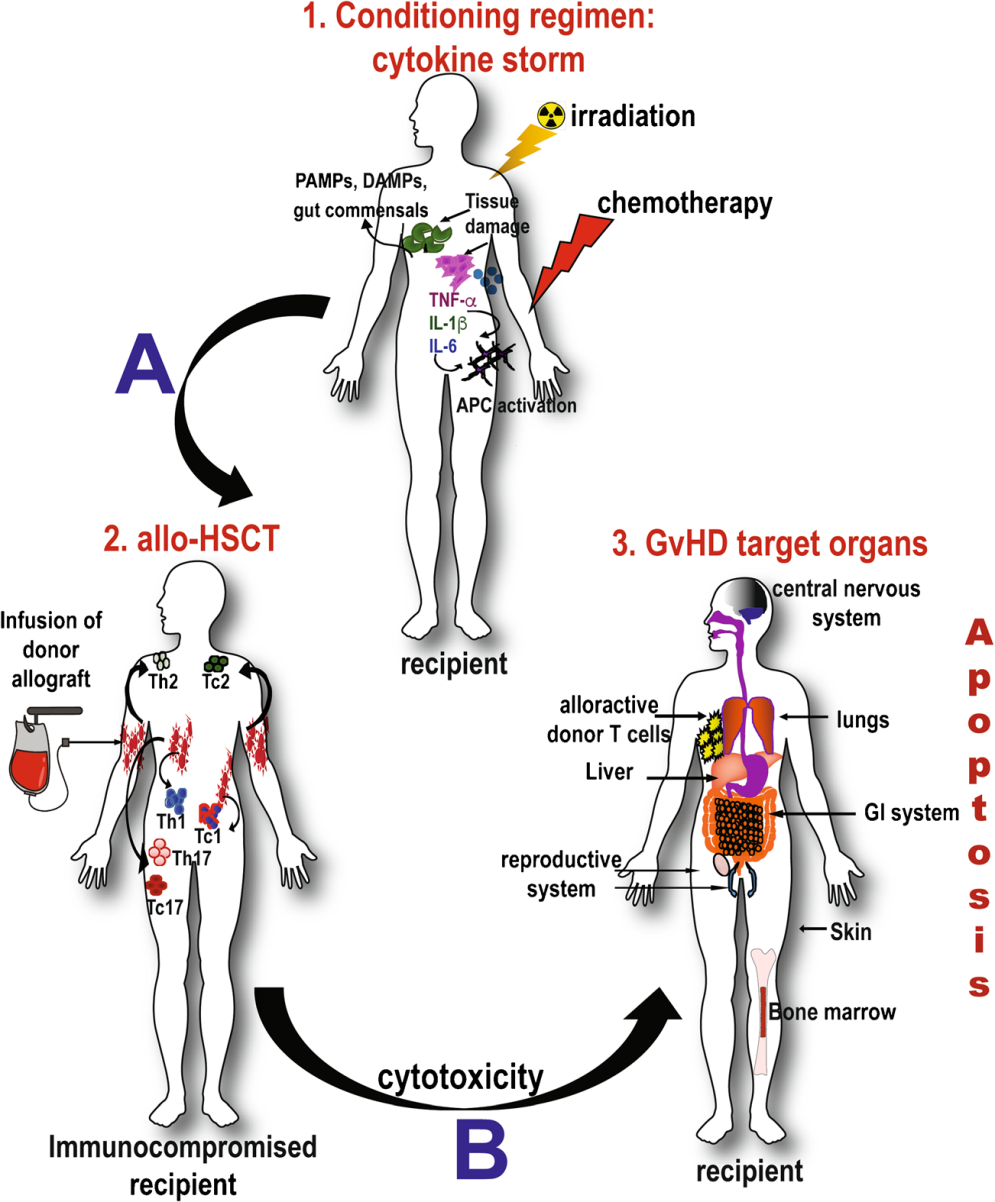
Development of GvHD. The onset of GvHD starts with the conditioning regimen, which involves irradiation and/or chemotherapy. This regimen produces tissue damage and the concomitant “cytokine storm” characterized by the release of pro-inflammatory cytokines such as IL-1β, IL-6, and TNF-α, as well as damage-associated molecular patterns (DAMPs) and pathogen-associated molecular patterns (PAMPs). These danger signals activate host antigen-presenting cells (APCs). The immunocompromised patient then undergoes allogeneic-hematopoietic stem cell transplantation (allo-HSCT). The host-activated APCs then also activates the proliferation and polarization of allo-reactive donor T cells, including Th1/Th2/Th17 for CD4^+^ Tc1/Tc2/Tc17 for CD8^+^ T cells, which ultimately induce the development of GvHD. These activated pathogenic T cells infiltrate multiple target organs including the gut, the central nervous system, liver, tract, skin, and the reproductive system, amplifying local tissue destruction, for example, via apoptosis and other cellular dysregulations

For many malignant and nonmalignant hematological or immunological diseases, the only cure can be acquired in combination with allogenic hematopoietic stem cell transplantation (allo-HSCT) [1]. The most favorable disease-free response rates after allo-HSCT have been observed in patients with chronic myeloid leukemia (CML), several lymphomas, multiple myeloma (MM), and acute myelogenous leukemia (AML). However, the benefit of this type of immune-rescue transplantation is limited by suitable HLA-matched donor availability. In fact, less than 25–30% of eligible patients actually acquire human leukocyte antigen (HLA)-matched donors reviewed by [2]. In addition to this, allo-HSCT generally results in a high incidence of the life-threatening complication graft-vs-host disease (GvHD), which is the cause of considerable morbidity and mortality. The onset of GvHD is mediated by allogenic donor-derived immune cells, especially allo-reactive T lymphocytes that upon transplantation become activated by mismatched major and/or minor histocompatibility complex antigens within multiple tissues of the recipient. As a result, a cascade of molecular events involving antigen-induced proliferation and differentiation of donor T cells and the release of pro-inflammatory cytokines by these activated T cells ultimately target and damage numerous organs and tissues in the transplant recipient, including the liver, lungs, ovary, or testis, central nervous system, gut, and skin [2] (Fig. 1).

In an effort to prevent induction of GvHD, or ameliorate its pathologic consequences, non-myeloablative and reduced conditioning regimens along with prophylactic drugs have been used with partial beneficial outcomes. Despite the significant progress achieved during the last decade to prevent, or overcome the severity of, GvHD after allo-HSCT, it remains the major cause of non-relapsing cancer mortality, being fatal for up to 15–20% of recipients undergoing allo-HSCT [3]. Therefore, novel and innovative therapies are urgently required in order to improve both the cancer-free and GvHD-managed outcomes for patients receiving allo-HSCT. This review will cover standard as well as novel and innovative emerging therapies for preventing, or treating GvHD, without compromissing the positive benefits of graft-versus-tumor (GvT) effects. In particular we focus on exploiting adjunct ex vivo virotherapy of the allo-HSCT sample prior to transplantation.

### Pathophysiology of Graft-Vs-Host Disease

GvHD occurs when donor T cells activate and respond to HLA mismatches on recipient’s tissue. Three stages contribute to the onset of aGvHD [4]. First, tissue damage occurs after conditioning regimen, which in turn activates host antigen presenting cells (APCs). The second stage is called the afferent phase. In this phase, host APCs activate donor T cells leading to GvHD. Donor T cells can recognize host antigens (Ags) on host APCs, primarily by direct antigen presentation [5]. As an alternative host Ag presentation can be mediated by donor APCs, which present host Ags to donor T cells via an indirect pathway of antigen presentation predominantly via major histocompatibility complex (MHC) Class II to CD4 T cells. In this regard, it was demonstrated that conventional DCs (cDCs) are the primary APC s responsible for alloantigen presentation after allogeneic bone marrow transplantation [6]. It has been proposed that the interruption of the process by which the host alloantigen is presented to donor T cells to generate GvHD could be a new therapeutic strategy to prevent the morbidity and mortality followed after allo-transplantation. The third stage is called an efferent phase, in which cellular and inflammatory factors contribute to the protracted damage of target organs [4]. Based on the time frame and the type of organ involvement, GvHD occurs in two distinct forms, acute and chronic GvHD (aGvHD and cGvHD, respectively) [2]. Table 1 summarizes immunological and clinical differences between aGvHD and cGvHD.

**Table 1:** Differences between aGvHD vs. cGvHD

	aGvHD	cGvHD
Onset	</ = 100 days following allo-HSCT	> 100 days following allo-HSCT
Risk factors	Recipient and donor ages, HLA-gender-disparity, multiparous female donors, ineffective GvHD prophylaxis, and intensity regimen [4]. Furthermore, damage of the intestinal epithelium causes release of bacteria and alteration in the gut microbiome. This triggers the prolonged activation of the immune system and the subsequent amplification and severity of aGvHD [5, 6]. Besides bacteria, *Candida* colonization of gut is also a risk factor of aGvHD [6].	Acute GvHD (aGvHD), recipient and donor ages, the type of donor, intensity of conditioning regimen, the source of the stem cells, in vivo depletion of T cells (using antibodies such as alemtuzumab or anti-thymocyte), sex mismatch, HLA disparity, race, and previous infection with cytomegalovirus or Epstein Barr virus [7, 8].
Overview of the GvHD pathophysiology	Acute GvHD (aGvHD) is primarily driven by activation of donor T cells by host alloantigens and the induction of pro-inflammatory cytokine storm [6, 9]. The onset and development of aGvHD occurs in 3 phases: Phase 1: Tissue damage from ablative or non-myeloablative conditioning regimens. Phase 2: Donor T cell activation mediated by host APCs (afferent phase). Phase 3: T cell proliferation, secretion of the inflammatory effectors IL-1, IL-6, and TNF, adhesion molecules, and upregulation of MHC antigens [9]. This is called the efferent phase. In addition to T cells, gastrointestinal (GI) tract damage and subsequent alteration of the intestinal homeostasis also play a role in the exacerbation of aGvHD. During GvHD, intestinal stem cells (ISCs) and secreted Paneth cells, which have a role in tissue renewal and regeneration of injured epithelium, are not recovered. In addition to Paneth cells, goblet cells help maintain the intestinal microbial ecology and protect hosts from pathogens, reviewed by [6].	The pathology of cGvHD involves multiple and distinct interactions among allo-reactive and dysregulated T and B cells and innate immune populations, including macrophages, dendritic cells (DCs), and neutrophils [7]. The initiation and development of cGVHD involve 3 phases: Phase 1: Inflammation and tissue injury, orchestrated by the activation of the innate immune system, which recruits Th1/Tc1 and Th17 cells to the tissue site. Release of soluble cytokines, toll-like receptor (TLR) agonists in response to cytotoxic agents and aGvHD. Secretion of chemokines in response to IFN-α and IFN-γ. Phase 2: Involves chronic inflammation and dysregulation immunity as a result of activation of donor adaptive immune T and B cells, antigen presenting cells (APCs), and NK cells. Phase 3: Involves propagation of tissue injury by dysregulation of multiple lymphocyte subpopulations. This results in the release of profibrotic mediators that induce the activation of macrophage and fibroblasts, fibrosis, collagen deposition, and irreversible organ dysfunction [7].
Target organs	Skin, gastrointestinal tract, liver, central nervous system, and ovary [6].	Lung, skin, liver, intestinal tract genital tissues, esophagus, musculoskeletal, joint, facial, ocular, and oral organs [10].

The pathogenesis of GvHD is ascribed to the tissue-destructive activities of the donor leukocytes, in particular the activated T lymphocytes. In the setting of an allogeneic HSCT, in which the donor transplant sample is usually either derived from bone marrow or mobilized PBMCs, the T cells in the donor graft are activated upon interaction between donor T cell receptors (TCRs) and major (or minor) histocompatibility complex (MHC)-bound host allo-antigens, resulting in vigorous T proliferation, differentiation, and migration into multiple host target organs and tissues [11] (Fig. 1). Furthermore, allo-reactive T cells mediate the hyper-activation of many stimulatory immune pathways, cytokine signaling, perpetuating the severity of GvHD. Immune effectors like cytokines are known for regulating both innate inflammation and acquired cellular immunity, and in the setting of allo-HSCT, an over production of many cytokines by activated T cells and other inflammatory cells contributes to the clinical manifestations of GvHD [2]. Table 2 describes the role of some of these pro-inflammatory cytokines and chemokines in the onset and severity of GvHD (Tables 1 and 2). In addition to cytokines and chemokines, toll-like receptors (TLRs) and the nucleotide binding oligomerization domain (NOD)-like receptors (NLRs) are key components of the innate immunity. TLRs/NLRs regulates the APCs’ activities via cytokine and chemokine release and phagocytosis, antigen presentation [15]. Table 2 briefly summarizes the role of TLRs/NLRs innate immune receptors in the outcome and occurrence of GVHD.

**Table 2:** The role of cytokines/chemokines and other immune molecules in the pathogenesis of GvHD

Pre-transplant and conditioning regimen derived cytokines [12, 13]	Role in GvHD
Tumor necrosis factor alpha (TNFα) and Interleukin 1 (IL-1)	Conditioning regimen including chemotherapy, radiotherapy, or both produce host tissue damage especially the intestinal mucosa. This promotes the translocation of microbial lipopolysaccharide (LPS) from the intestinal lumen to the circulation, stimulating the secretion of TNFα and IL-1 from host macrophages. These cytokines activate host antigen presenting cells (APCs), as well as increase the expression of major histocompatibility antigens (MHC-Ags) and adhesion molecules on host tissues, which in turn augment the recognition of MHCs and minor histocompatibility antigens (mHAgs) by mature donor T cells. Therefore, these proinflammatory cytokines contribute to the gut GvHD pathogenesis and increase the morbidity and mortality related to GvHD.
Interleukin 6 (IL-6)	IL-6 like IL-1 and TNFα, produces tissue damage. IL-6 is produced by B cells, mononuclear cells and skin keratinocytes. This latter is a target of GvHD. Increase production of this cytokine by the skin during GvHD produces exacerbation of the disease. In the presence of IL-2, IL-6 induces the differentiation of T cells into cytotoxic T cells. IL-6 also synergizes with IL-3 to promote differentiation and maturation of hematopoietic stem cells (HSCs) and maturation of megakaryocytes to platelets. IL-6 is involved in all phases of GvHD.
Th1 derived cytokines [11, 13]	Role in GvHD
Interleukin-2 (IL-2)	IL-2 is implicated in activation, proliferation and expansion of T cells during GvHD. The role of IL-2 in GvHD involves the amplification of the allogeneic immune response, activation of T cells, NK cells and the secretion of TNF-α by macrophages. High dose of IL-2 after allo-HSCT attenuates GvHD mortality in irradiated mice. However, low dose of IL-2 decreases the incidence of GvHD. Importantly low IL-2 restores the homeostasis of regulatory T cells (Tregs) without impairing the GVT effects. However, there is some controversy in the use of IL-2 to suppress GvHD. For example, in an experimental mouse GvHD model administration of IL-2 to a donor mouse induces proliferation of Tregs but is insufficient to suppress GvHD. In a xenogenic mouse model of GvHD, low-dose of IL-2 increased Tregs but it did not control the production of proinflamatory cytokines by conventional T cells (Tcons)
Interleukin-12 (IL-12)	Donor T cell activation in phase 2 of GvHD is characterized by the presence of IL-12. IL-12 is a heterodimeric cytokine produced by DCs and macrophages that mediates cellular immunity. The dimeric components are the subunits p40 and p35. Because subunit p40 drives Th1 differentiation the use of anti-p40 Ab appears to reduce aGvHD.
Interferon-gamma (IFN-γ)	IFN-γ is important in both innate and adaptive immune responses, as well as in the induction and regulation of antimicrobial, antiviral and anti-tumor immunity. IFN-γ is produced by activated T cells, NKT cells and NK cells. IFN-γ inhibits GvHD in lethally irradiated mice undergoing allo-HSCT. However, in sub-lethally, or un-irradiated mice IFN-γ increases the lethality of GvHD. Therapeutic effects of IFN-γ appear to depend on the conditioning regimen.
Th2-derived cytokines [11, 13]	Role in GvHD
Interleukin-3 (IL-3)	IL-3 is involved in the differentiation and apoptosis of several hematopoietic cells. Expression of IL-3 is upregulated in patients with cGvHD.
Interleukin-4 (IL-4)	The pleiotropic cytokine IL-4 is produced by activate T cells, and play a key role in regulation, or pathogenesis of allogeneic responses.
Interleukin-5 (IL-5)	IL-5 triggers differentiation of activated B cells. High levels of IL-5 are observed in the serum of patients with aGvHD.
Interleukin-10 (IL-10)	IL-10 inhibits T cell proliferative responses and proinflammatory cytokine synthesis. IL-10 Is a regulatory cytokine that modulates CD4^+^ T cells by downregulating IL-2. IL-10 doesn’t contribute to GvHD mediated by effector T cells. In contrast, IL-10 generates a tolerogenic environment to alloantigens independent of IL-2 or CD28 stimulation. The induction of IL-10 in host B cells attenuates GvHD. However, low frequency of IL-10 responses increases the severity of GvHD.
Interleukin-13 (IL-13)	IL-13 plays a role in inflammatory diseases like GvHD. For example, pre-transplant of serum IL-13 has been correlated with the severity of GvHD. In fact, mixed leukocyte reaction (MLR) supernatants and skin explant assay of GvHD correlates higher levels of IL-13 with GvHD.
Th17-derived cytokines [11]	Role in GvHD
Interleukin-17 (IL-17)	IL-17 is produced by both CD4^+^ and CD8^+^ T cells. IL-17 is abundant in the serum of patients with GvHD and is associated with mortality.
Interleukin-22 (IL-22)	IL-22 protects intestinal stem cells from immune-mediated tissue damage. IL-22 producing retinoic acid-related orphan receptor and type 3 innate immune cells (RORγτ^+^ILC3^+^) appears to prevent intestinal GvHD. *In vivo* studies have shown that treatment with IL-22 after allo-HSCT enhanced the recovery of intestinal stem cells, increased epithelial regeneration and reduced mortality associated with GvHD.
Interleukin-21 (IL-21)	IL-21 is involved in GvHD development through increasing B cell activation and proliferation, generation of alloantigen and disrupting the Tregs homeostasis. Inhibition of IL-21 decreased the severity of GvHD symptoms.
Other cytokines and chemokines reviewed by [11]	Role in GvHD
Interleukin-35 (IL-35)	IL-35 is an anti-inflammatory cytokine that can suppress GvHD in patients receiving allo-HSCT. IL-35 targets and up-regulates the phosphorylation of STAT1 and STAT4, which is inhibited in murine models with aGvHD. Thus IL-35 treatment ameliorates aGvHD in mice.
Interleukin-7 (IL-7) and Interleukin-15 (IL-15)	IL-7 and IL-15 are homeostatic cytokines with a dual role in promoting lymphocyte reconstitution in mice and humans, and in aGVHD following allo-HSCT. During GvHD, high systemic levels of IL-7 and IL-15 have been associated with aGvHD development after myeloablative transplant [14].
B cell activating factor (BAFF), IL-33, CXCL10 and CXCL11	Increased levels of these cytokines and chemokines are part of the pathogenicity of GvHD. It is controversial that binding of IL-33 to the receptor called suppression of tumorigenicity 2 (ST2) results in both proinflammatory and anti-inflammatory effects. ST2 is a secreted biomarker of refractory GvHD. The blockade the IL-33 and ST2 interaction reduces the lethality of GvHD.
CCR7	In mesenteric lymph nodes of the gastrointestinal (GI) tract, CCR7 regulates elevated alloantigen presentation. Thus CCR7 has been associated with GI complications during GvHD.
CD103	The expression of transforming growth factor 1 beta (TGF1-β)-dependent CD103 regulates the destruction of gut epithelium by CD8^+^ T cells during GvHD.
IL-1β	Upon conditioning regimen uric acid and microbial products activate the inflammasome protein called nucleotide-binding domain and leucine-rich repeat 3 (NLRP3) in donor T cells, which in turns increases the expression of IL-1β. High levels of IL-1β then enhances the severity of GvHD.
Innate immune receptors: toll-like receptors (TLRs) and TLR ligands [15]	Role in the outcome of GvHD
TLR4	Because TLRs control the adaptive immune response, it has been hypothesized that TLR signals influence the activation of donor T lymphocytes and exacerbate the outcome of GvHD. In this regard, LPS a ligand of TLR4 mediates the activation of this receptor, which leads to the release of proinflammatory cytokines. In particular, TLR4 mediates severity of GvHD in the GI tract.
TRR7/8	TLR7/8 are expressed on plasmacytoid dendritic cells (pDCs), which are anti-viral APCs. pDCs express the immunosuppressive enzyme indoleamine 2,3-dioxygenase (IDO), which influence the pathology of GvHD. Administration of TLR7/8 agonist induced the production of IDO and the decrease of GvHD severity [15].
The TLR5 ligand flagellin	The TLR5 agonist protein flagellin modulates the innate and adaptive immunity in mice and humans. In addition to this, flagellin protects epithelial cells from toxicity post-radiation. Flagellin help maintain gut immune homeostasis [16]. Flagellin reduces cGvHD in patients receiving allo-HSCT. In fact, flagellin suppresses the APC function and favors the generation of immune suppressive Tregs, reviewed by [17].
TLR9	CpG DNA is an agonist of TLR9 (CpG is a DNA region where a cytosine nucleotide is followed by a guanine). It has been shown that TLR9 ligation of APCs by the CpG DNA increases the mortality associated with GvHD in a murine transplantation model. TLR9 and its downstream adaptor myeloid differentiation primary response gene 88 (MyD88) play a role in the immunopathology observed in a murine intestinal GvHD model [17].
Nucleotide binding oligomerization domain (NOD)-like Receptors (NLRs) [15]	Role in GvHD occurrence
NOD2	NOD2 contributes to the susceptibility to GvHD after allogeneic-HSCT. In contrast to TLRs, the absence of NOD2 from the mouse donor bone marrow (BM) allograft did not regulate alloactivation of donor T cells, with no impact in the development of GvHD. However, deficiency of NOD2 in the BM transplant recipients increased the incidence of GvHD in both MHC-matched and MHC-mismatched models.

### GvHD Prevention and Treatment

Current prophylaxis and treatment for GvHD are partially effective with high risk of disease relapse, development of infections, and long-term adverse effects. Because the centerpiece of prophylaxis and treatment of GvHD involve T cell manipulation, different approaches have been used in clinical trials to prevent or treat GvHD. Table 3 summarizes common pharmacologic drugs used for the prophylaxis of GvHD.

**Table 3:** Prophylaxis and/or treatment of GvHD

Standard pharmacologic drugs	Function	Advantages and/or disadvantages
Calcineurin inhibitors (CNIs): cyclosporine A (CsA), tacrolimus (FK506) [18]	Calcineurin is an activator of nuclear factor of activated cell (NFATc) that catalyzes some intracellular processes associated with the activation of T cells. CNIs inhibit calcineurin, which results reduced production of IL-2, prevention of T-cell activation, and differentiation [19].	In pediatric patients, the use of CNI alone prevents the severity of GvHD [20]. In general, CNIs produce nephrotoxicity causing late renal allograft loss. Calcineurin induces the release of endothelin-1 a potent vasoconstrictor
Methotrexate (MTX)	MTX is an anti-metabolite and an analog of aminopterin, the folic acid antagonist. At low doses, methotrexate attenuates the activation of T cells [21].	High doses of MTX are associated with toxicity of kidneys, gastrointestinal (GI), mucosa, and liver [22]. Hematopoiesis delays [18] engraftment and increases infection risk [20]. MTX in combination with CsA has improved the prophylaxis of GvHD.
Mycophenolate mofetil (MMF)	An anti-metabolite and prodrug of mycophenolic acid, which selectivity inhibits inosine monophosphate dehydrogenase in T cells. Combination of MMF with any calcineurin inhibitors has shown synergistic activity in the prophylaxis of GvHD [23].	The use of MMF is exclusive after non-myeloablative and cord transplants [24].
Sirolimus (SIR)	SIR binds to the intracellular protein FKBP12 inhibiting the mammalian target of rapamycin (mTOR) pathway and blocking IL-2-mediated signaling. This leads to cell cycle arrest in naïve T cells. Furthermore, SIR has permissive effect on the thymic generation of regulatory T cells allowing the expansion of them [25].	Because SIR has anti-neoplastic and antiviral properties, inhibits maturation of dendritic cells (DCs), and carries low toxicities, it is an attractive immunosuppressant agent for GvHD prophylaxis. However, SIR alone, or in combination with CNIs, is very toxic after intensive conditioning reviewed by [26].
Standard and emerging approaches to prevent or treat GvHD		
Targeting allo-reactive T cells	Function	Advantages and/or disadvantages
Anti-thymocyte globulin (ATG) and alemtuzumab	Both ATG and alemtuzumab are anti-lymphocytic antibodies that suppress the reaction of host T cells enabling engraftment. In addition, both contribute to eliminate donor T cells [27].	Reduces the risk of GvHD, but attenuates the benefits GvT effects. Moreover, the recovery of lymphocytes post-transplant is hindered by these Abs, resulting in a higher risk of opportunistic infections [27].
Inhibition of protein kinase C isoforms θ and α (PKCθ and PKCα, respectively.)	- Inhibition of the activation of T cells. - Deletion of PKCθ partially blocks TCR signals and ameliorates GvHD. PKCα provides activation of IL-2 feedback. Cooperation of PKCθ with PKCα is required for T cell signaling and function.	In murine models of allo-HSCT, the inhibition of PKCθ and PKCα impaired donor T cell proliferation, migration, and chemokine/cytokine production. Also, the pharmacologic inhibition of PKCα/θ with R524 spared the T cell cytoyoxic function and the GvT effects [28].
Visilizumab (Abbottt), a monoclonal antibody against CD3.	Visilizumab a humanized monoclonal anti-CD3 Ab has a T cell receptor partial agonist ligand function induces selective apoptosis of activated T cells [29].	Safety and biological activity has been reported in clinical studies of glucocorticoid-resistant GvHD treatment, reviewed by [29]. Although the use of anti-CD3 Abs halts active autoimmunity in murine models, these Abs are less effective in preventing GvHD [29].
Bortezomib (BOR)	BOR is a selective proteasome inhibitor [30]. BOR prevents the activation of NF-κB, which is highly expressed in activated T cells. BOR targets and induces apoptosis of activated T cells and reduces the levels of IFN-γ and IL-2, [31]	Adverse effects include risk of complications, secondary infections, delay of the immune system reconstitution, graft rejection, and cancer relapse.
Statins	Statins have immunomodulatory effects, including the inhibition of antigen-presenting cells [32]. The use of Lovastatin, for example, inhibits the secretion IL-2, IL-4, and IFN-γ from primary human activated T cells [33]. Lovastatin also impairs the proliferation of donor T cells post-transplant and thus, the severity of GvHD [34].	One limitation is that the statin-associated GvHD protection was restricted to recipients co-treated with cyclosporine post-allograft engraftment [35]. On the other hand, in recipients with CSA-based post-graft immunosuppression, the use of statin at the time of allo-HSCT was associated with a decrease of cGvHD risk, but with increased risk of cancer relapse [36].

### Emerging Approaches to Prevent or Treat GvHD

Besides targeting, or manipulating allo-reactive T cells, other approaches to prevent GvHD are focused on induction of regulatory T cells, targeting B cells, mesenchymal stem cells (MSCs), and the use of chemokine and cytokine antagonists such as maraviroc, TNF inhibitor, IL-2 receptor antagonist, and IL-6 inhibitor. In this section, we briefly discuss some of the more relevant emerging approaches used to abrogate GvHD.

### Therapy with Regulatory T Cells Against GvHD

Regulatory T cells (Tregs) are CD4^+^CD25^+^FoxP3^+^ cells that promote immunotolerance and play a key role in inhibiting excessive immune responses, and preventing, or delaying rejection of the allografts [37]. Tregs suppress early expansion of allo-reactive T cells and decrease the capacity to induce GvHD without impairing the GvT effects [38]. Although several clinical trials have demonstrated the safety and efficacy of Tregs to reduce aGvHD, major challenges that face this therapy include difficulties to obtain pure Tregs and the limitation to manufacturing Tregs in large scale. In the setting of cGvHD, low numbers of circulating Tregs are observed. Because the homeostasis of CD4^+^ Tregs is maintained by IL-2, administration of low doses of IL-2 increased the proliferation of Tregs and increased the generation of thymic Tregs [39, 40]. Thus, stimulation of Tregs with low dosage of IL-2 seems to contribute to the suppression of cGvHD.

### B Cell Targeting Strategies

Increasing evidence links B lymphocytes with the pathogenesis of aGvHD and cGvHD [41]. There are growing evidences suggesting that B cells contribute to the immune response via the production of pro-inflammatory cytokines, antigen presentation, and other immunoregulatory functions, as reviewed elsewhere [42]. Also, dysregulation of germinal center (GC) B cell formation results in the secretion of pathologic antibodies that are implicated in fibrotic tissue destruction [41]. Early after HSCT, elevated levels of B cell activation factor (BAFF) along with increase of allo-antigens provide optimal environment for B cell receptor activation and pathologic antibody formation. Inhibition of mature B cells with anti-CD20 partially prevents cGvHD in humans [41]. In a different study, the CD20-blockade with rituximab, a chimeric murine/human anti-CD20 monoclonal antibody, appears to reduce the incidence and severity of acute GvHD following allogeneic HSCT [42, 43]. Thus, a better understanding of B cell dysregulation may have an impact on limiting the severity of GvHD.

### Mesenchymal Stem Cells

Mesenchymal stem cells (MSCs) are non-hematopoietic pluripotent progenitor cells that are found in adult BM as well as in adipose and perinatal tissues [44]. MSCs support hematopoiesis and can attenuate diverse immune reactions. For example, MSCs do not elicit T-cell responses in vitro [45]. Moreover, in experiments involving a mixed lymphocyte reaction (MLR), human MSCs did not stimulate allo-PBMCs, or lymphocyte proliferation [46]. Thus, in the setting of allo-HSCT, these cells seem attractive for infusing patients regardless of the HLA-matching. In a clinical study, a 9-year-old child with acute lymphoblastic leukemia (ALL) that had received allo-transplantation, with an HLA-A HLA-B, HLA-DRβ1 identical and unrelated female donor, developed grade IV aGvHD at day 70 post-transplant. He then received ex vivo–expanded MSCs from his mother with remarkable improvement [47]. More recently, Kim et al. reported that infusion of MSCs primed by IFN-γ into NOD/SCID mice reduced the symptoms of GvHD and increased survival rates compared to infusion of naïve MSCs [48]. The authors found that expression of indoleamine 2,3-dioxygenase (IDO) by MSCs is required in order to see the beneficial effects. The expression of IDO in MSCs occurs via the IFN-γ-Janus kinase (JAK)-signal transducer and activator of transcription 1 (STAT1) (IFN-γ-JAK-STAT1) pathway. The expression of IDO was then correlated with the suppression of T cell proliferation [48].

Because MSCs support stem cell engraftment, inhibit lymphocyte responses, and are safe post-infusion, MSCs have emerged as potential treatment of complications related to allogeneic HSCT [49]. In the setting of HLA-mismatched transplantation, a reduction of GvHD was observed when MSCs were co-infused with the allo-transplant. However, in patients co-transplanted with HLA-matched identical siblings HCT plus MSCs, GvHD was reduced but a higher incidence of relapsed disease was observed [50].

### Viruses and GvHD

Recent studies have demonstrated both negative pathologic consequences and positive anti-cancer benefits that can accompany cytomegalovirus (CMV) reactivation in terms of relapse of myeloid malignancies following allo-HSCT [51]. However, the reactivation of CMV is also associated with non-relapse mortality (NRM) as a result of opportunistic infection after grades II to IV acute graft-vs-host disease (aGvHD) [51]. Cytomegalovirus infection is an important complication for patients receiving allo-HSCT [52, 53]. More than 26 years ago, Riddell et al. demonstrated that virus-specific T cells from a healthy donor could be generated ex vivo from autologous CMV-infected fibroblasts. Importantly, when these T cells were adoptively transferred to recipients undergoing allo-HSCT, prevention of CMV infection and prevention of GvHD were observed [50].

In a different approach, allogeneic transplantation of donor lymphocytes engineered with the suicide gene thymidine kinase of herpes-simplex virus (TK) showed therapeutic potential to control GvHD. However, this methodology has been restricted due to the difficulty to ex vivo manipulation of donor lymphocytes and the limitation in the generation of the TK^+^ cells, which require rigorous conditions to upscale these TK^+^ cells under good manufacturing practice (GMP) conditions.

### Oncolytic Viruses

Because a variety of neoplasms remain incurable with current standard therapies, novel and innovative anti-cancer therapies are required in order to sustain long-term cancer regression and prolong patients’ lives. Both immunotherapy and oncolytic virotherapy are new promising approaches to clinical cancer therapeutics. The success of oncolytic viruses (OV) in the clinical setting depends on the selective tumor cell oncolysis by the therapeutic virus, followed by activation of cellular immune responses against both viral and tumor antigens. One common characteristic of many candidate OVs is that they are safe for normal healthy cells and tissues, but selectively infect and replicate in a wide spectrum of human cancer cells [55]. Myxoma virus (MYXV), a DNA virus, is a preclinical candidate OV that belongs to the *Poxviridae* family. In nature, MYXV exhibits a highly restricted host range and is only pathogenic to European rabbits. Importantly, it has been shown that MYXV can also infect a wide variety of human cancers, including pancreatic, ovarian, melanoma, glioblastoma, and various hematologic malignancies such as MM and AML. Preclinical studies have also demonstrated that MYXV is a safe OV candidate even in highly immunodeficient mice [56]. MYXV is being currently developed to be used as either an anti-cancer monotherapy or as an adjunct virotherapeutic in combination with current standard therapies like HSCT, or coupled with emerging immunotherapies to treat different types of cancers. In this section, we briefly discuss the state of the art of oncolytic virotherapy, with special emphasis on MYXV as a potential adjunct therapy for allo-HSCT.

There are at least 2 doz viruses that are now in the path to be translated form the bench to the bedside, including measles virus, vesicular stomatitis virus, adenovirus, reovirus, herpes simplex virus, parvoviruses, and two poxviruses, vaccinia virus, and MYXV [57–62]. Vaccinia virus has been widely used as a vaccination platform against smallpox, and recently, this virus has been tested as oncolytic virotherapeutic in phase II clinical trials for liver cancer [57, 63]. In 2015, talimogene laherparepvec (a.k.a. T-VEC), an oncolytic herpes simplex virus, became the first oncolytic virus to be approved by the FDA to treat metastatic melanoma [64].

### Oncolytic Virotherapy for Hematological Malignancies

The use of OVs has garnered considerable interest as cancer therapeutics and is currently under intense clinical investigation. Among different hematologic malignancies, multiple myeloma (MM) has begun to emerge as a prime candidate for oncolytic virotherapy. MM is a clonal plasma cell (PC) malignancy with an estimated of 30,770 new cases and 12,770 patient deaths in 2018 [65]. Despite significant progress in the prognosis of MM, overall survival rates are still modest with less than 50% of patients surviving 5 years, as reviewed elsewhere [66]. Stem cell rescue following high doses of chemotherapy with autologous HSCT is the standard therapy for younger patients with MM. However, minimal residual disease (MRD) and/or contaminating tumor cells within the autograft, leading to disease relapse, is the major drawback of auto-HSCT. Therefore, novel strategies are urgently required in order to improve MM-free patients.

Of the many viruses that are currently under investigation for MM are the RNA viruses including measles virus, vesicular stomatitis virus, reovirus, and coxsackievirus 21 and DNA viruses such as adenovirus, vaccinia virus, and MYXV. To date, MYXV is the only OV shown to be capable of both ameliorating GvHD following allo-HSCT, in addition to possessing anti-cancer activities. However, to put this seemingly unique feature of MYXV in perspective, we discuss this virus in context with other OVs being currently developed against various hematologic malignancies that are currently eligible for HSCT.

## Oncolytic RNA Viruses

Measles virus (MV), a negative-strand RNA virus that belongs to the genus *Morbillivirus* under the family *Paramyxoviridae*, causes infections in the respiratory tract. Virus entry to the cells occurs through the interaction of the viral hemagglutinin-glycoprotein (H-glycoprotein) with the CD46 receptor, which is overexpressed in cancer cells like MM [67]. Edmonston-B vaccine strain (MV-Edm) is a replicating virus that has been attenuated after repetitive tissue culture passage [66]. Earlier in vitro and in vivo preclinical studies with the live attenuated MV-Edm (specifically, GFP-tagged MV-Edm) demonstrated effective lysis of MM cells in vitro as well in MM patients with no adverse effects in normal lymphocytes. Likewise, human tumor cells implanted in SCID/NOD murine xenograft model showed completed tumor regression following intratumoral treatment, or systemic delivery of MV [68]. Other MV-Edm derivatives utilizing human carcinoembryonic antigen (CEA; MV-CEA) or human sodium iodide symporter (NIS; MV-NIS) have been used in order to improve the delivery efficiency of the virus to sites of MM, as reviewed elsewhere [66]. The MV-NIS in particular has shown a remarkable therapeutic effect against MM xenografts [69]. The preclinical efficacy and the safety data generated from the MV-NIS have contributed to translate this virus to phase I clinical trial for recurrent or refractory MM [70]. Because patients receiving allo-HSCT are more susceptible to virus infection, including measles virus infection, in the setting of cGvHD, vaccination against this and other opportunistic viruses is always required [71]. This makes MV a less-likely candidate as virotherapeutic adjuvant in the setting of allo-HSCT.

Reovirus is a non-enveloped double-strand RNA virus with minimal pathogenicity in humans [72]. Reovirus is internalized into cells via the ubiquitous sialic acid receptor [73] and/or the junction adhesion molecule (JAM) [74]. In order to infect and kill target cells, this virus usurps activated signaling pathways, such as Ras, of cancer cells [73]. In vitro, in vivo, and ex vivo studies have showed that reovirus exhibits oncolytic activity against a variety of solid neoplasms including prostate, ovarian, colorectal, breast, and gliomas as well as hematologic malignancies such as chronic lymphocytic leukemia (CLL), non-Hodgkin’s lymphoma, and MM [66, 75–80]. Notably for this discussion, in vitro and in vivo studies have shown evidence that this oncolytic virus does not harm hematopoietic stem cells or their colony-forming activities [81]. Preclinical studies have explored using reovirus to delete contaminating MM cancer cells from samples used for autologous stem cell transplantation (ASCT) [76].

Vesicular stomatitis virus (VSV) is a negative-strand-enveloped RNA virus that belongs to the *Rhabdoviridae* family. The virus has the ability to cause vesicular lesions in farm animals [83]. However, the incidence of human infection is rare [84], and when infection occurs, this is generally benign [85]. Because VSV is sensitive to interferon (IFN), the virus exploits IFN-dysregulated pathways of tumor cells for its replication [86]. Preclinical studies have shown that MM and several leukemic cell lines and ex vivo patient’s samples are very sensitive to VSV variants V1 and V2 and the heat resistant (HR) VSV. VSV variants have minimal effect on colony-forming ability of hematopoietic stem cells, suggesting the potential use of these VSV mutants as a cancer cell purging agent for autologous HSCT samples [87, 88]. The VSV∆51 variant was engineered to express the human sodium iodide symporter (hNIS) for combined imaging and radiotherapy of MM. The VSVΔ51-hNIS showed oncolytic properties against MM cell lines as well as primary patient tumors, producing high titers in MM cells under in vitro conditions. Infusion of VSV∆51 to bg/nd/xid mice model bearing subcutaneous myeloma tumors resulted in cancer regression and high intratumoral virus replication [88].

Coxsackievirus 21 (CVA21) is a non-enveloped positive-strand RNA that belongs to the *Picornaviridae* family. In humans, this virus causes myositis and respiratory tract infections [89, 90]. However, CVA21 exhibits oncolytic potential against MM [91]. CVA21 infection and oncolysis of MM cells required the receptors intracellular adhesion molecule-1 (ICAM-1) and decay-accelerating factor (DAF), which are both overexpressed in MM cells [91, 92]. Preclinical studies showed that CVA21 infects RPMI 8226, U266, and NCI-H929 MM cell lines. Importantly, normal human PBMCs were resistant to virus infection [91]. Incubation of CVA21 with primary bone marrow samples derived from patients with MM resulted in virus purging of the malignant MM CD138^+^ plasma cells at up to 98.7% with minimal effects in progenitor cell function [91]. Because CVA21 can cause severe myositis in immunocompromised mice, a micro-RNA approach was used to decrease pathogenicity of the virus [93]. Immunocompromised SCID mice bearing subcutaneous Kas 6/1 MM tumor cells were treated with muscle-specific miRNA inserted into the CVA21 virus in order to decrease the pathogenicity of the virus by destabilizing virus replication in a tissue-specific manner. As a result, complete regression of MM cells was observed without any signs of myositis [93]. Despite the promising data, CVA21 has not yet been translated into clinical trials as an oncolytic virotherapeutic agent against MM.

## Oncolytic DNA Viruses

Adenovirus (Ad) members are non-enveloped double-stranded DNA viruses, and wild-type Ad may cause mild clinical infections of the upper respiratory tract; however, they may also cause significant morbidity and mortality in immune-compromised patients [66]. Attenuated adenoviral vectors have been studied in the majority of studies involving Ad as an oncolytic agent. In children, Ad infection is cause of morbidity and mortality after allo-HSCT [94]. In contrast, the incidence of Ad infection in adult patients is lower [95]. After allo-HSCT, incidence of infections due to Ad is observed in all the phases of the procedure, including pre-engraftment, early post-engraftment, and late phases post-engraftment [96].

Preclinical studies showed the efficacy of Ad to deliver the thymidine kinase (TK) gene into MM cell lines like OCI-My5 and RPMI 8226, as well as primary patient samples [97]. Because MM cancer cell lines and primary MM samples express the adenovirus receptor coxsackievirus and adenovirus receptor (CAR), the carcinoma-selective protein DF3/MUC1 and the integrins *α*_v_ *β*_5_ or *α*_v_ *β*_3_ are required for the internalization of the virus [98]; it was logical to use Ads that selectively deliver genes under the control of the DF3 promoter. Teoh et al. showed that transduction of Ad bearing the TK gene under the control of the DF3 promoter (Ad.DF3-NK) followed by treatment with 50 μmol/L of ganciclovir (GCV), an anti-viral drug used to treat cytomegalovirus infections, deleted more than 6 logs of contaminated OCI-My5- and RPMI 8226–contaminated bone marrow mononuclear cells. Importantly, normal human hematopoietic cells were not affected under these treatment conditions [97]. In a study using a conditionally replicating Ad carrying a CD40 ligand transgene (AdEHCD40L), the authors showed a potentiated growth inhibition of MM cells [99]. In effect, AdEHCD40L-mediated apoptosis was observed in MM susceptible cell lines. In vivo studies performed by the same group, using a SCID xenograft mouse model pre-implanted with RPMI 8226 and then treated with AdEHCD40L, showed a 50% decrease in MM as compared to controls (e.g., 28% MM tumor reduction) [99]. Despite the promising data derived from experiments using Ad as an oncolytic agent against hematologic malignancies like MM, a major concern for a clinical use of this oncolytic virus is its high immunogenicity with the concomitant induction of a strong immune response in the host and the high levels of anti-Ad sero-reactivity in human populations [100]. In the setting of allogeneic HSCT, infection rates with human adenovirus approach 5–21% in transplant patients [101, 102]. The overall human Ad-associated mortality ranges from 18 to 26% [103] and mortality rates of 14–100% in infected patients, regardless of any virostatic treatment as described by Matthes-Martinet et al. [104]. In addition, the administration of at least some anti-viral drugs is associated with nephro- and myelo-toxicity [102]. Therefore, it seems very unlikely that this OV would be used to treat hematologic malignancies in conjunction with allo-HSCT.

Vaccinia virus (VACV) belongs to the *Poxviridae* family and is a close relative of the smallpox virus [105]. VACV is a double-stranded DNA virus with a large genome of 190 Kb. Vaccinia virus exhibits strong immunogenicity, resulting in high T cell responses and circulating antibodies, and was used as the vaccine to eradicate smallpox in the 1970s [106]. Different VACV strains have been investigated as oncolytic virotherapeutic agents [106, 107]. McCart et al. developed the first oncolytic double gene knockout–attenuated VACV, in which the VACV TK gene and the vaccinia growth factor (VGF) genes were both deleted and an enhanced green fluorescent protein (EGFP) was inserted at the TK locus (VACVDD-GFP) [108]. This attenuated VACV showed oncolytic activity against MM cell lines as well as primary samples from patients with MM and minimal virus infectivity [109]. In addition, mice bearing human subcutaneous OCI-My5 or disseminated RPMI 8226 MM cells that were treated with VACV showed increased survival and decreased tumor burden compared to untreated controls [109].

The first clinical study using VACV dates back to 1987 when a 67-year-old patient with IgA MM received intravenous injection of VACV Ankara strain (AS) resulting in the decrease of the levels of IgA from 1309 mg/dL in the early stage of the treatment to 432 mg/dL on the day 96 of the regimen and with no adverse effects [110]. In 2008, Park and co-workers reported the mutant VACV JX-594, which selectively replicated in, and eliminated, metastatic liver cancer [111]. The TK-deleted, human granulocyte-macrophage colony-stimulating factor (hGM-CSF)-armed JK-594 (a.k.a. Pexa-Vec) selectively infects and kills cancer cells with cell cycle abnormalities and epidermal growth factor receptor/Ras-aberrant signaling pathways [111]. Pexa-Vec is in phase II clinical trials to treat patients with hepatocellular carcinoma [112]. Preclinical experiments revealed that MM cells are susceptible to VACV infection [113]. However, one of the major clinical issues is the virus dose-dependent toxicity of normal tissues. Up to now, VACV has not been tested in conjunction with HSCT for any hematologic malignancies.

Myxoma virus (MYXV) is a member of the *Poxviridae* family whose natural tropism is restrictive to rabbits. There are no reported anti-MYXV antibodies in any human populations. Like vaccinia, MYXV is a double-stranded DNA poxvirus with its replication cycle strictly performed in the cytoplasm. Notably, MYXV is nonpathogenic to humans or any non-rabbit vertebrate hosts, including mice or any domestic animals. Studies performed in our lab have shown that MYXV had oncolytic activity against a wide variety of human cancer cells in vitro and in vivo [114]. In 2009, Kim et al. reported that MYXV selectively infects and kills primary human leukemia cells (AML) while sparing normal hematopoietic stem and progenitor cells used for immune rescue transplantation [115]. Although some notable exceptions exist of cells to which MYXV cannot bind, the virus binds to, and can initiate infection of, most mammalian cells. However, the virus can discriminate between permissive (e.g., rabbit cells or most human cancer cells) or non-permissive (e.g., primary human leukocytes) cells by virtue of their endogenous signaling pathways [116]. For example, activation of the AKT signaling pathway by constitutive AKT phosphorylation, or induced by MYXV infection, regulates permissiveness of this virus to different human solid tumor cell lines [117]. In another study, the pro-inflammatory cytokines such as TNF and type I IFN are induced upon infection of normal human macrophages generating an anti-viral response that results in MYXV infection abort in these non-permissive cells [118]. It is known that cancer cell defects in the TNF/IFN-signaling pathway are common and that cancer cells defective of this signaling pathway become more susceptible to MYXV infection [119]. Likewise, different human cancers show excessive levels of activated AKT, thus facilitating the replication of MYXV [117].

In 2012, studies performed by Bartee et al. revealed that infection of human MM cell lines with MYXV resulted in the efficient eradication of these MM cancer cells via induction of rapid cellular apoptosis, whereas normal human hematopoietic and progenitor cells (CD34^+^) were spared by the virus [120]. In addition, ex vivo treatment of human MM cells with MYXV prevented the subsequent engraftment of these cells into an immunocompromised NOD/Scid/IL2Rγ^−/−^ (NSG) host. Importantly, MYXV did not compromise the engraftment of HSPCs from the CD34^+^ compartment because of the inability of MYXV to bind or infect this cell population [120]. In fact, it is the safety of MYXV for immune engraftment by CD34^+^ HSPCs that allows the virus to be used as an adjunct ex vivo therapy for either auto- or allo-HSCT. In addition, intravenous (i.v.) systemic delivery of MYXV into BALB/c mice bearing the murine MOPC315 myeloma cell line eliminates these cancer cells via induction of rapid cellular apoptosis mediated by the systemic MYXV treatment [121].

More recently, our lab has explored primary leukocytes, such as T cells and neutrophils, as “cell carriers” of MYXV to deliver the virus to sites of disseminated cancer following HSCT. Published results have demonstrated that MYXV can hitchhike on both murine C57BL/6 T cells and neutrophils from bone marrow to ferry the virus to, and eliminate, disseminated MOPC315.BM.DsRed MM cells in MHC-mismatched BALB/c recipient mice [122]. This study indicates that ex vivo virotherapy of allo-HSCT bone marrow samples with MYXV improves disease-free survival rates of recipients bearing pre-seeded MM. Similar studies to test ex vivo MYXV virotherapy as an adjuvant for autologous HSCT against pre-seeded MM are in progress.

### MYXV as Potential Adjunct Therapy for GvHD

High-dose chemotherapy followed by autologous HSCT is considered the standard of care for newly diagnosed patients with certain hematological malignancies, such as MM. Nevertheless, a vast majority of myeloma patients die from the disease as a result of the recrudescence of minimal residual disease (MRD) that had presumably persisted within disease niches at the time of transplant and/or the re-infusion of myeloma cells that can contaminate the autograft. In order to improve disease-free responses and overall survival, tandem approaches including autologous transplantation, non-myeloablative allogeneic transplants, post-transplant maintenance, and immunotherapy strategies have been tested ([119], evolving options for MM). Even though the standard treatment for MM is with autologous HSCT, in some cases depending on donor availability and the patient’s age, allogeneic stem cell transplantation has been used less frequently to treat MM [120]. However, a wider application of allogeneic transplantation for MM is limited, in part, because the high median age of patients diagnosed with MM is 63 years [119]. A combination of high-dose therapy and autologous HSCT for the reduction of tumor burden followed by low-intensity conditioning and infusion of allogeneic-stem cells as immunotherapy was proposed in 2003 for newly diagnosed MM patients [121]. One of the major challenges with MM is the presence of residual MM cells in disease niches that resist elimination by standard therapeutic regimens. One alternative to treat minimal residual disease after autologous HSCT is with the tandem therapy described above. Tandem autologous, followed by reduced-intensity allograft performed in 120 patients reported 18–24% mortalities related with the transplant. The occurrence of cGvHD was 7–60% and survivals ranged from 58 to 74% at 2 years, 86% at 3 years, and 69% at 5 years reviewed by [12]. In another study, 114 patients received autologous HSCT after conditioning regimen with melphalal or bursufal-mephalal. Patients with no available donors (88 patients) received a second autologous HSCT following treatment with etoposide, cyclophosphamide, and carmustine. On the other hand, 26 patients received a reduced-conditioning allograft after a regimen with melphalal and fludarabine. The transplant-related mortality was 5–16% for the group receiving the tandem auto-HSCT and 11–33% for the tandem auto-HSCT and allo-HSCT. The allografts were favored over the auto-HSCT in terms of event-free survival [122]. Therefore, adjunctive strategies to improve allo-HSCT are required in order to increase the efficacy and safety of this therapy for cancers like MM. In addition to this, novel strategies that prevent or minimize GvHD need to be explored in clinical trials.

Recently, several convergent lines of preclinical evidence have suggested that ex vivo virotherapy of human HSCT samples with MYXV can ameliorate the onset of GvHD, while maintaining or enhancing the beneficial GvT effects in the setting of allo-transplantation against MM. First, in vivo experiments using a xenograft (human-to-murine) model demonstrated that human bone marrow samples pre-treated ex vivo with MYXV and then transplanted into NSG mice greatly reduced mortality compared with control engrafted mice (e.g., human BM samples that were not treated with the virus) [128]. Death of the control mice transplanted with human bone marrow was attributed to xeno-aGvHD caused by expansion and activation of human donor CD3^+^ T cells in multiple internal organs of the recipient mice. In contrast, ex vivo treatment of the donor human BM with MYXV prevented the development of GvHD, and histological analyses of the recipient internal organs revealed them to be mostly free of donor lymphocytes [128]. Authors from these in vivo studies concluded that ex vivo MYXV treatment reduced the severity of post-transplant GvHD caused by xeno-geneic stem cell transplants by interfering with the ability of the donor human T cells to induce aGvHD. In a subsequent study, we reported that MYXV was able to inhibit the development of GvHD after xeno-HSCT in this model because the virus efficiently binds to resting human CD3^+^ T lymphocytes but aborts at this early stage, whereas following T cell activation, the virus block is relieved in a fashion that launches the full virus replication cycle and at the same time impairs the functionality of the activated human T lymphocytes [129]. Thus, although MYXV binds to resting human T cells found within an HSCT sample, only after these T cells receive a cell activation signal (e.g., with anti-CD3/CD28 antibodies in vitro or by contact with allo-antigen in vivo), the T cells now become productively infected with this oncolytic virus. This productive infection of primary human T cells results in them not only becoming virus “carrier cells” but also causes the inhibition of T cell proliferation and decreased expression of GvHD-promoting cytokines such as IL-2, IL-2Rα, and IFN-γ [126]. High levels of these pro-inflammatory cytokines are associated with the severity of GvHD following allo-HSCT [127]. Furthermore, MYXV-infected/activated T cells could deliver MYXV to human MM cancer cell line (U266) via cell-cell contact, resulting in the infection and further killing of these cancer cells [129]. Thus, MYXV-augmented T cells could target and kill MM via a virus-vs-tumor (VvT) effect, as well as an enhanced graft-vs-tumor (GvT) effect, or likely a combination of both VvT and GvT. Therefore, in the context of allo-HSCT, ex vivo virotherapy with MYXV seems to have a dual role not only inhibiting aGvHD but also enhancing GvT effects against residual MM.

**Fig. 2:**
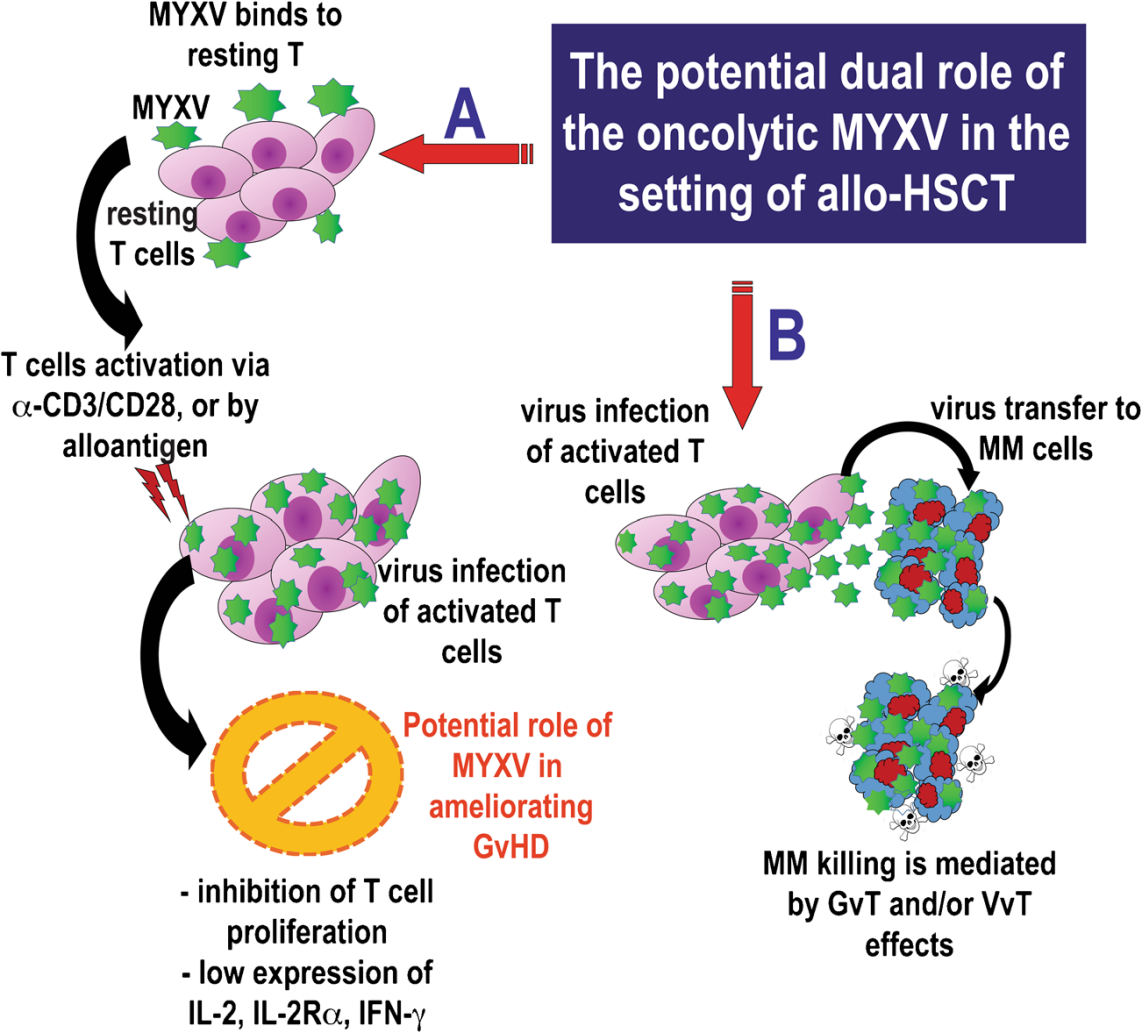
The potential dual role of the oncolytic MYXV in the setting of allo-HSCT. The oncolytic virus MYXV can bind efficiently to resting human T cells but the virus infection halts at this early stage. However, following activation of T cells via α-CD3/CD28 stimuli or by contact with an alloantigen, these activated cells now launch the full virus infection cycle and become virus-bearing “carrier cells.” The productive infection of activated T cells also impairs T cell functions including their capacity to proliferate and downregulates the expression of at least some of their GvHD-promoting effector cytokines. For example, activated human T cells infected with MYXV produce lower levels of IL-2, IL2-R-α, and IFN-γ, which are part of the hallmark of GvHD. In a related scenario, when MYXV-infected/activated human T cells are co-cultured with human MM U266 cancer cell line, the virus (either parental or progeny) can be transferred to these cancer cells via cell-cell contact. Once contacted, the myeloma cells are eliminated via GvT (graft-vs-tumor) and/or VvT (virus-vs-tumor), or a combination of both

More recently, in vivo experiments using a classic mismatched mouse to mouse allogeneic HSCT model (e.g., donor C57BL/6 bone marrow transplanted into BALB/c recipient bearing pre-seeded mouse MM) confirmed the oncolytic potential of MYXV to decrease tumor burden and increase the survival rates in the recipient mice [122]. Although for this in vivo study the primarily focus was to investigate the optimal delivery strategy for MYXV (e.g., systemic delivery using virus-loaded murine carrier cells from the transplant sample, such as T cells or neutrophils, to deliver MYXV to disseminated sites of MM such as in the bone marrow and spleen) as a model for minimal residual disease, it is likely that GvHD was also involved after the allo-transplantation [122]. This study was focused on the tumor burden declines and the increased survival rates observed in the immunocompromised and MHC-mismatched recipients transplanted with allogeneic BM preloaded with MYXV, and the severity of GvHD in these recipient mice cohorts was not specifically assessed. Therefore, further investigation is required in order to clarify if MYXV alone or as an adjunct therapy can contribute to the inhibition of GvHD in vivo following a mouse-to-mouse allo-transplant. Figure 2 summarizes our findings using the oncolytic MYXV to ameliorate GvHD while maintaining or enhancing the anti-cancer benefits of GvT.

As mentioned before, the standard treatment for MM is with myeloablative therapy along with autologous HSCT. However, to a lesser extent, some MM patients have also been treated with reduced-intensity conditioning followed by allo-HSCT [130]. Thus, the possibility of using MYXV in the context of allo-HSCT as adjunct therapy against GvHD for any hematological cancer patient receiving allo-HSCT remains a tantalizing possibility. Thus, it is imperative to expand and improve our knowledge regarding the molecular mechanisms used by MYXV to inhibit or control GvHD. A better understanding of how MYXV abrogates GvHD would also open new possibilities for other cancer patients whose disease can only be treated with standard therapies along with allo-HSCT.

## Conclusion and Future Perspectives

Up to now, MYXV is the first and the only oncolytic virus that has been reported to eliminate residual cancer (e.g., MM) and also prevent GvHD in the setting of allo-HSCT. It is possible that other select oncolytic viruses might also possess these dual anti-cancer/anti-GvHD properties, as long as it could be demonstrated that (1) the virus cannot infect primary CD34^+^ hematopoietic stem cells and is harmless for immune engraftment, (2) the virus can target, infect, and eliminate a spectrum of hematological cancers in patients who are eligible for HSCT, (3) the virus can load onto other primary leukocytes within the allo-transplant sample and migrate with these cells to sites of disseminated cancer in the transplant recipient, and finally, (4) the virus can infect and compromise the effector functions of CD3^+^ T lymphocytes that normally become activated and drive GvHD following allo-HSCT.

Undoubtedly, a better understanding of GvHD biology will pave the way to develop novel treatment strategies with improved clinical benefits for patients receiving allogeneic HSCT. However, despite the myriad of biological and pharmacologic therapies used in combination with allogeneic transplantation procedures, GvHD still represents the major cause of morbidity and mortality of non-relapsed malignancies. In addition, the treatment of some very aggressive cancers like MM is mainly restrictive conventional therapies or autologous HSCT. However, although these therapies often do provide years of additional life, they still do not induce long-term survival in the majority of patients with MM. Combination therapies, including tandem treatments (e.g., chemotherapy, auto-HSCT, low-conditioning allo-HSCT), are attractive but their application is restrictive to those patients that have matched MHC-related or unrelated donors. In addition, more extensive research is required in order to improve the efficacy of these tandem therapies. A new innovative strategy is the use of oncolytic viruses like MYXV in the setting of allo-HSCT. Recent studies have shown that MYXV could have a dual role not only inhibiting the development of GvHD but also maintaining or enhancing the positive benefits of GvT effects derived from allo-HSCT. However, more preclinical studies are required in order to understand the molecular mechanism used by MYXV to control allo-reactive T cells and to improve the effector mechanisms of GvT.
